# Radiology resident education during the COVID-19 pandemic in the United States

**DOI:** 10.3389/fmed.2025.1595754

**Published:** 2025-06-17

**Authors:** Mohammed I. Quraishi, Alan Itkin, Alexandria Atkins, Benjamin Davis, Robert Eric Heidel, Mark Andrew Rowley, Ethan Nussio, Richard Gray

**Affiliations:** Department of Radiology, University of Tennessee Medical Center, Knoxville, TN, United States

**Keywords:** radiology, residency, COVID-19, education, learning, competence

## Abstract

The COVID-19 pandemic presented unprecedented challenges to healthcare systems, including radiology residency programs. This study aims to examine the impact of COVID-19 on radiology residency education and identify interventions implemented for future unplanned disruptions to physician training. Data collection occurred between March to April 2022 through a survey distributed to 30 radiology residency program directors from diverse geographic regions, hospital types, and practice settings. Data was collected on program characteristics, COVID-19 impact, changes in scheduling and teaching methods, and perceived effects on resident competence and well-being. All surveyed programs implemented changes to address resident teaching to accommodate social distancing. Most programs (86.7%) offered remote work/study options. A majority (66.7%) implemented alternating resident schedules. Virtual conferences and virtual view-box teaching were identified as the most utilized interventions during social distancing requirements. The majority (76.1%) of programs reported worsened resident education during the pandemic, with first-year residents the most adversely affected group. Decreased competence was noted in 40% of first-year and 36.7% of second-year residents compared to pre-pandemic cohorts. Additionally, a significant portion (73.3%) of program directors reported negative impacts on resident well-being. The COVID-19 pandemic significantly disrupted radiology residency despite mitigation efforts. While virtual teaching methods provided necessary alternatives during the pandemic, they could not fully replace traditional in-person education, as evidence by widespread reports of worsened educational outcomes. Recommendations for future preparedness include prioritizing early deployment of remote workstations, incorporating alternative teaching methods, providing increased on-site instruction for junior residents, and enhancing mental health support. These lessons can inform strategies to better prepare residency programs for future challenges and ensure the continued production of competent, resilient radiologists.

## Introduction

During the coronavirus disease of 2019 (COVID-19) pandemic, the healthcare system faced unprecedented challenges, with frequently changing governmental policies and guidelines. One sector of the healthcare system that was strongly impacted was resident education, including diagnostic radiology. The volume of imaging interpretation by residents during the pre-pandemic to intra-pandemic time period was reduced by 62.8% (1). Reduction in training volume resulted in more than 40% of radiologists reporting worry their professional activity could be damaged (2). Moreover, the pandemic shifted the education of residents to an online format to follow social distancing guidelines and reduce the risk of exposure to disease, utilizing virtual instruction methods such as view-box teaching and virtual conferences. A majority (53–74%) of experienced radiology residents perceived this virtual learning to be less effective than in-person training (3). Resident programs also shifted to a hybrid format to limit exposure during the pandemic and follow social distancing guidelines. This hybrid formation had a negative effect on resident productivity, with an estimated 32% fewer studies being interpreted by residents (4). The changes made to radiology resident education during the pandemic had both positive and negative effects. This study aims to examine the impact of COVID-19 on radiology residency education and identify interventions implemented by programs during the pandemic to inform future preparedness strategies for unplanned disruptions to physician training.

## Materials and methods

A survey was distributed to 195 radiology residency program directors within the United States from varying geographic regions, with representation from both community and academic hospitals, as well as private and hospital-owned groups. Data collection was conducted between March 15, 2022 to April 15, 2022, approximately 2 years after the initial pandemic disruption in March 2020. Study data were collected and managed using Research Electronic Data Capture (REDCap), a secure, web-based software platform designed to support data capture for research studies (5, 6). Thirty programs responded to the survey (response rate: 15.3%). The demographic information of the programs is included in Table 1. Questions included in the survey asked programs about number of residents and attendings diagnosed with COVID-19, the timing of when changes were implemented into the residency program, whether remote work options were provided, scheduling changes, alternative educational activities such as virtual lectures, and whether any of these changes were deemed to be useful and might be carried forward following the pandemic. Other questions included which year of residents were most affected and whether these residents differed in competence compared to previously graduated residents who did not experience a pandemic.

**Table 1:** Demographics of the surveyed radiology residency programs.

Region	*N* (%)
Region	
South	10 (33.3)
Northeast	9 (30.0)
Midwest	8 (26.7)
West	3 (10.0)
Practice type	
Hospital-owned	24 (80)
Private practice	6 (20)
Hospital type	
Academic	22 (73.3)
Community	8 (26.7)

## Results

### Characteristics of programs affected and timing of change implementation

Responses were obtained from programs within each geographic region, reflecting the distribution of programs within the United States (Table 1). The number of residents per program varied from 5 to 44, with an average of 22.6 residents (SD ± 10). The median number of residents was 22.5. 40% of program directors believed that their pre-pandemic workload could be handled comfortably without residents (resident-independent programs). 60% of programs did not believe they could cover the workload without residents (resident-dependent programs).

During the pandemic’s height, a majority of programs reported faculty and resident illness (Table 2). The average percentage of residents who were diagnosed with COVID prior to the first vaccine rollouts was 7.7% (SD ± 10.3%). Programs reporting any resident having COVID-19 represented 73.3% of the sample, while programs reporting no sick residents represented 26.7% of the sample. For faculty/staff, 90.0% of programs reported someone having COVID-19, and 10% reported no sick faculty/staff.

**Table 2:** Percentages of residents, faculty, and staff diagnosed with COVID-19.

Program reporting of COVID-19	Diagnoses
Residents diagnosed with COVID-19 prior to first vaccine rollout (*N*)	7.7 (SD 10.3)
Programs with any resident diagnosed with COVID-19 (%)	73.3
Programs reporting no residents diagnosed with COVID-19 (%)	26.7
Programs with any faculty/staff diagnosed with COVID-19 (%)	90.0
Programs with no faculty/staff diagnosed with COVID-19 (%)	10.0

All programs made changes to address resident teaching during social distancing guidelines. A majority (25; 83.3%) of the programs reported making changes in March 2020 and the remaining 5 programs (16.7%) made changes in April 2020. A higher percentage of resident-independent programs made this change in March (91.7%) than resident-dependent programs (77.8%).

### Changes related to scheduling and preventing infection

Table 3 summarizes the various changes programs made during the pandemic to minimize the infection risk for residents and faculty. A majority (86.7%) of programs offered remote work/study options for residents at the height of the pandemic. However, only a minority (26.7%) of programs reported availability of off-site workstations. 19 programs (63.6%) offered either unguided self-study at home or guided self-study at home. Only 7 programs (23.3%) offered research as a remote work/study option. Alternating resident schedules were implemented by 20 programs (66.7%). Most programs (60%) enacted a schedule where only half of the residents were in-house at any one time. This was either done weekly, biweekly, and with one program reporting having a morning shift and an afternoon shift. Other responses are seen in Table 4.

**Table 3:** Strategies employed by programs to minimize infection risk and continue resident education.

Strategy	%
Remote work/study option	86.7
Availability of off-site radiology workstations (%)	26.7
Guided or unguided self-study (%)	63.6
Remote research (%)	23.3
Alternating resident schedules (%)	66.7

**Table 4:** Scheduling changes made by residency programs to maximize social distancing but ensure adequate staffing.

Alternative resident scheduling
Rotating half the residents
Two-thirds on-site and one-third remote
Staggering schedules
Ensuring trauma center staffing
Minimal on-site group (“skeleton crew”)

### Changes related to teaching methods, education, and wellness

All programs reported virtual noon/morning conferences being implemented during the pandemic. 73.3% of programs reported implementing “virtual view box teaching” using programs such as Microsoft Teams screen sharing and 73.3% of programs reported “teaching over the phone.” Only half of the programs required residents to complete a question bank. A large minority (43.4%) implemented teaching files (Table 5). A majority (90%) of programs reported other educational activities being implemented during the pandemic. By far, virtual view-box teaching and virtual conferences were perceived as the best educational value while social distancing by 90% of programs (27/30). A vast majority of responding programs (93.3%) stated these new educational methods will be used even after the end of the pandemic.

**Table 5:** Various methods related to teaching residents employed by programs during the pandemic.

Teaching method	%
Virtual conferences (%)	100
Virtual view box teaching (%)	73.3
Teaching over the phone (%)	73.3
Required question bank (%)	50.0
Virtual teaching cases (%)	43.4

A majority of the responding programs (76.1%) stated that resident education during the pandemic worsened. 13.3% felt that education did not change and 6.7% felt education improved (Table 6). Faculty size decreased at 36.7% of programs during the pandemic and 67% of programs affected reported significant negative impacts on resident education. The majority of respondents (60%) indicated that the first-year radiology class was the most adversely affected by the pandemic. Specifically, when asked how each class compared to their immediate predecessors who were not affected by the pandemic a large minority saw decreased competence in the first year and second year radiology residents (Table 7).

**Table 6:** Program directors’ perceptions of the impact of COVID-19 and loss of faculty on the quality of resident education.

Impact of COVID-19 on resident education	%
Impact of COVID-19 on resident education	
Worsened (%)	76.1
No change (%)	13.3
Improved (%)	6.7
Impact of reduced faculty size during pandemic on resident education	
Worsened (%)	67

**Table 7:** Program directors’ impressions of resident competence by class year compared to their immediate predecessors who were not affected by the pandemic.

Class year	Less	Unchanged	More
R1 (%)	40	56.7	3.3
R2 (%)	36.7	63.3	
R3 (%)	20	76.7	3.3
R4 (%)	10	83.3	6.7

20% of program directors felt their 2021 graduates were less competent than previous years. Given program directors are privy to In-Service exam scores and ABR pass rates for their residents, an objective question was asked about these metrics. 26.7% of program directors reported decreased In-Service exam scores compared to 2020. 30% of program directors reported decreased ABR pass rates in 2021 compared to pass rates prior to the pandemic. 53.3% of program directors reported decreased resident engagement in scholarly activities (Table 8).

**Table 8:** Radiology residency programs reporting negative effects of the pandemic on graduate competence, exam scores, and resident engagement.

Effect of COVID-19 on resident performance and competence	%
Less competence in graduates compared to prior years (%)	20
Decreased In-Service exam scores (%)	26.7
Decreased board pass rate (%)	30
Decreased resident engagement in scholarly activities (%)	53.3

Program directors were asked how residents’ well-being was affected by the pandemic on a 5-point Likert scale (Table 9). The results are as follows: Significantly Worsened = 13.3%, Worsened = 60.0%, Unchanged = 23.3%, and Improved = 3.3%. Program directors were asked about wellness activities during the pandemic, the most common answers were zoom social hour (100%), wellness outings (96.7%), increased resident lunches (90%), small gatherings outside hospital (83%), and outdoor activities (76.7%).

**Table 9:** Program directors’ impressions of the impact of the pandemic on resident well-being.

Program impression on resident well-being	%
Significantly Worsened (%)	13.3
Worsened (%)	60.0
Unchanged (%)	23.3
Improved (%)	3.3

## Discussion

The COVID-19 pandemic presented unprecedented challenges to radiology residency programs, impacting both the educational experiences of residents and their well-being. Our analysis of residency programs across the country underscores several key findings and recommendations for future preparedness.

During the height of the pandemic, programs faced challenges related to faculty and resident illness, with a substantial proportion reporting COVID-19 cases among residents and faculty. This underscores the importance of implementing measures to ensure the safety and well-being of all individuals involved in residency training. Program directors’ perceptions regarding workload dependence on residents varied, with 40% of programs considered resident-independent and 60% resident-dependent. This distinction may have influenced the strategies adopted by programs in response to the pandemic, particularly regarding changes in resident teaching and scheduling. It should be noted that all programs made changes to resident schedules to decrease the number of in-house hours with the vast majority making changes early in the pandemic demonstrating good faith efforts to protect residents.

The shift toward remote work options during the pandemic were primarily study-oriented and revealed the need for greater access to remote clinical experiences for residents. Clinical work is the root of radiology education and perhaps if off-site workstations were deployed for residents there would have been less academic blunting. There was also a substantial loss of faculty, decreasing the faculty-to-trainee ratio, which was likely a large driver in decreased quality of resident education. Future planning should prioritize the early deployment of remote workstations and access to clinical cases to provide a more comprehensive and balanced educational experience.

Virtual view-box teaching and virtual conferences emerged as the most commonly implemented interventions during social distancing requirements, with 100% of programs utilizing virtual conferences and 73.3% implementing virtual view-box teaching. While these methods provided necessary alternatives when in-person instruction was not possible, they could not fully compensate for traditional educational approaches. Despite widespread implementation of these virtual platforms, 76.1% of programs still reported worsened resident education during the pandemic. This suggests that while virtual teaching methods served as crucial stopgap measures, they have inherent limitations compared to traditional in-person training. The fact that 93.3% of programs plan to continue using these virtual platforms post-pandemic likely reflects their value as supplementary tools rather than primary educational methods. Technical difficulties and family obligations at home presented additional challenges that further limited the effectiveness of remote learning. Future preparedness should focus on optimizing these virtual methods as backup options while maintaining robust in-person training as the primary educational approach. However, despite these efforts, a significant proportion of program directors reported worsened resident education, decreased engagement in scholarly activities, and negative impacts on resident well-being, consistent with prior studies (3, 7). These findings highlight the multifaceted challenges faced by residency programs during the pandemic and the ongoing need for support systems and interventions to address these issues. Our analysis indicated a differential impact on resident competence, with lower-level residents being more affected by disruptions in training routines, this contrasts with Shi et al. which reported the third year-residents being the most disrupted of classes (8). We believe that younger residents were most impacted due to the reduced amount of in-person readouts and decreased number of cases to read (9, 10). Special consideration and increased on-site instruction for lower-level residents may help mitigate these challenges and ensure smooth maturation into the senior years. It is also crucial to address the impact of the pandemic on resident emotional well-being. Residency programs should prioritize mental health support and early professional intervention to ensure the overall well-being of trainees. It should be noted that there are limitations in our study. There is inherent recall bias as program directors were asked to retrospectively assess changes in resident education and competence over a rapidly evolving crisis, particularly given the timing of data collection 2 years after the initial pandemic disruption. Although program directors used In-Service exam scores and ABR pass rates, they also used subjective judgment to assess resident competence which introduces observer bias. The study has a response rate of 15.3% which limits its generalizability. Additionally, our assessment of resident well-being and educational impact relies on program director perceptions rather than direct resident feedback, which may not fully capture the residents’ experience.

While the COVID-19 pandemic posed significant challenges to radiology residency programs, it also provided valuable insights and opportunities for growth. Lessons learned from the pandemic experience can inform future preparedness strategies for residency programs, including optimizing remote learning infrastructure, enhancing resident support and wellness initiatives, and adapting educational curricula to ensure continued competence and engagement among residents (11). By implementing the recommendations outlined in this analysis, residency programs can better prepare for future catastrophic events and ultimately continue to produce competent and resilient radiologists no matter the challenges they face.

## Data Availability

The raw data supporting the conclusions of this article will be made available by the authors, without undue reservation.
